# Mindreading by body: incorporating mediolateral balance and mouse-tracking measures to examine the motor basis of adults’ false-belief tracking

**DOI:** 10.1098/rsos.221212

**Published:** 2023-05-24

**Authors:** Giovanni Zani, Stephen A. Butterfill, Jason Low

**Affiliations:** ^1^ School of Psychology, Victoria University of Wellington, Wellington 6140, New Zealand; ^2^ Department of Philosophy, University of Warwick, Coventry CV4 7AL, UK

**Keywords:** motor processing, mediolateral leaning, false-belief tracking, mouse-tracking

## Abstract

The role played by motor representations in tracking others’ belief-based actions remains unclear. In experiment 1, the dynamics of adults’ anticipatory mediolateral motor activity (leftwards–rightwards leaning on a balance board) as well as hand trajectories were measured as they attempted to help an agent who had a true or false belief about an object’s location. Participants’ leaning was influenced by the agent’s belief about the target’s location when the agent was free to act but not when she was motorically constrained. However, the hand trajectories participants produced to provide a response were not modulated by the other person’s beliefs. Therefore, we designed a simplified second experiment in which participants were instructed to click as fast as possible on the location of a target object. In experiment 2, mouse-movements deviated from an ideal direct path to the object location, with trajectories that were influenced by the location in which the agent falsely believed the object to be located. These experiments highlight that information about an agent’s false-belief can be mapped onto the motor system of a passive observer, and that there are situations in which the motor system plays an important role in accurate belief-tracking.

## 1. Introduction

When we observe another person performing an action, our motor system becomes active as if we are executing that action (e.g. [1,2]). This mechanism allows us to track others’ goals—the outcomes to which their actions are directed—and to predict their movements. Although actions can be coded at a low muscle-specific level which strictly reflect how those actions are carried out kinematically (e.g. [1,3]), our motor system can also—when actions are directed to a goal—compute the best way (based on the context) of doing something now to achieve something later (e.g. [4]). In this way, contextual information that is actual [5,6] or non-actual [7,8], perceived or imagined, can refine or drastically change how means-to-an-end actions are actively planned and performed, as well as how they are coded in the motor system of a passive observer. However, it is still unclear whether and to what extent an observer’s motor system can also accommodate information about others’ beliefs to generate fast and accurate behavioural expectations.

When we reach to grasp an object, we use a set of necessary bodily displacements, such as arm extension and hand aperture, but the way we move is also influenced by situational constraints, such as the location of (e.g. [9,10]), or how familiar we are with, that particular object (e.g. [11]). For instance, when we see that the handle of our cup is not facing us but the opposite direction, we reach for it with an unconventional movement to achieve a more comfortable posture later [6]. And the kind of information that the motor system accommodates is not limited to what we can visually access. When our goal is to grasp an object, our kinematics will be modulated when we hear a sound that is incongruent with the sound of the contact target (e.g. aluminium-sound when reaching for a paper object; [12,13]) or even when we smell a fruit that has a different size of the one we are reaching for (e.g. smelling a strawberry when reaching for an apple; [14]) (for a review of the multisensory aspects associated with action execution see [15]).

Adults can also automatically track the goal an observed action is directed towards regardless of the specific effector used [16,17] or the perceptual availability of the movements [4]. For example, adults watching someone wearing a miniaturized soccer shoe kicking a ball with the index finger show motor facilitation in their own index finger, but also in their leg [16]. That is, while the observer’s motor system resonates with the low-level movements involved in the action (i.e. kicking the ball with the finger), it also codes for aspects of that action that are symbolic although not perceptually available in the actual environment (i.e. a soccer kick typically requires a leg movement) [18,19].

Overall, non-actual environments evoked during motor-imagery, such as the ones that are triggered by a sound, a smell or a symbolic value, can inform the motor system. Although motor imagery is the internal rehearsal of movements without any overt movement, it is well established [20] that it is characterized by a similar neural activation that occurs when preparing [21] and executing [22,23] an action. Also, similar to how performing an action depends on contextual limitations, the efforts taken to think about an action also increase as a function of imagined movement constraints. For example, it takes more time to walk, but also to think about walking, on a narrower beam compared to a larger beam [7]. In other words, regardless of how things actually are (that is, regardless of the fact that in reality the thinker is not actually executing nor observing any action), the thinker’s motor system becomes active as if it is performing that action within the limitations of the non-actual environment. Then, it is possible to conjecture that non-actual environments specified by an agent’s belief-like state could also modulate the observer’s motorically grounded expectations [19]. For example, consider a situation in which an agent has last registered the targeted object where it is not anymore while a passive observer knows the object’s current location. In this case, the agent will plan and execute actions based on her non-actual environment, and she will reach-to-grasp the object in the wrong location. Accordingly, the observer’s motor system needs to process information about the agent’s belief-like state (incompatible with the current reality) to motorically code that the best way (from the agent’s point of view) to do something now is actually to go to the wrong location.

There is some initial support for the conjecture that belief-like states can be processed by an observer’s motor system. In their adaptation of the ball detection task [24], van der Wel and colleagues [25] showed that analysing participants’ hand movements can be helpful to investigate how conflicts between one’s own and others’ beliefs are resolved automatically and online. Here, participants moved their mouse to click the location of a target ball with hand trajectories that were attracted towards the alternative empty location when the agent had a false belief about the whereabouts of the ball, regardless of the fact that the agent’s belief was irrelevant for the task. Recently, Zani *et al.* [26], used a Wii balance board (WBB) to study adults’ mediolateral leaning during the observation of live actions performed by an agent who had a false belief about the location of an object. They found that participants’ early motor activity foreshadowed the agent’s action, which was based on the non-actual environment as specified by her false belief. Indeed, before the agent displayed any overt cues to suggest which box she would have moved towards and regardless of the fact that participants were not instructed to track the agent’s beliefs, they spontaneously leaned in anticipation towards the box that was empty but that the agent believed to contain the object.

Considering that motor processes occur spontaneously and have a minimal impact on cognitive resources [27,28], Zani and colleagues’ [26] findings raise the possibility that it is useful for social cognition that motor representations of an upcoming action can consider others’ belief-like states to generate accurate behavioural expectations. There are, however, limitations to Zani *et al.*’s study. First, the live non-computer-based setting of their task set-up meant that they could not rule out the possibility that there may have been variability in the agent’s behaviour across participants. Consequently, for our current research, we created a computer-based application of Zani *et al.*’s [26] real-time interaction task to ensure consistency in presentation of the agent’s actions across participants. Second, the use of a balance board for answering questions about the motor-generated behavioural expectations underpinning belief tracking remains preliminary, and we note that even van der Wel and colleagues’ [25] mouse-tracking approach to study belief tracking is still not common in the theory-of-mind field. Given concerns that implicit or spontaneous measures for studying belief tracking may be fragile and difficult to replicate [29,30], we sought to determine if there is coherence between adults’ anticipatory mediolateral leaning and hand movements as they attempted to help an agent who had a false or a true belief about an object’s location. With respect to the different indicators of early action understanding, our prediction was that spontaneous leaning and mouse cursor trajectories would cohere in response patterning, with both metrics foreshadowing prediction of the agent’s action rather than the observer’s action.

We were also motivated to uncover new information about the extent to which motor-related information is mapped onto belief tracking. There is evidence suggesting that, in scenarios not involving beliefs, the ability to generate motor representations of an action is impaired by bodily constraining the observer [31] or even the agent [32]. For instance, Liepelt and colleagues [32] instructed participants to lift their index or middle finger in response to a number stimulus presented between the index and middle finger of a photograph of an agent’s static hand. Participants’ reaction times were slower when the agent’s index and middle fingers were tied compared to when the agent’s fingers were free, and compared to when the constrained fingers were those not involved in the participant’s action (thumb and ring finger). In other words, although participants’ ability to move was not directly manipulated, the functioning of their own motor system was significantly disrupted during the observation of an irrelevant agent being unable to move. In stressing such findings, we do not claim that belief tracking relies exclusively on motor-related information. There may be multiple routes to belief tracking. Nonetheless, if the onlooker’s motor system is sometimes necessary for tracking others’ beliefs, we predicted that even the bodily constraining of an agent should disrupt observers’ ability—as detected in participants’ body posture and mouse cursor trajectories—to motorically represent belief-based actions.

## 2. Experiment 1

### 2.1. Method

#### 2.1.1. Participants

Fifty-nine right-handed adults were recruited for this experiment in exchange for course credit. Two participants were excluded owing to technical problems. The final sample size was of 57 participants (*M* = 19.4 years, range = 18–31 years, 33 females and 24 males)

#### 2.1.2. Design and stimuli

Participants were tested individually in a single experimental session lasting approximately 1 h. They were asked to stand on a WBB with the right hand holding a mouse and the left-hand comfortably resting on the table. As per instructions, they watched video clips presented on a monitor in front of them and unlocked one of two boxes by clicking on it every time that the agent asked for help. The helping component was inspired by Buttelmann *et al.*’s real-time false-belief helping task [33]. The original authors were interested in how infants and young children help an agent who is unsuccessfully trying to open either a box in which she falsely believes the object is located or a box which she truly believes is empty. Buttelmann and colleagues reason that infants and young children in the false belief condition help the agent in opening the box containing the object because they understand that she wants to retrieve the object but has a false belief about its location. In the true belief condition they help the agent with the empty box she is directly struggling with because they understand that the agent is not trying to retrieve the object. Our overarching prediction focused on the different indicators of early behavioural expectation. To this end, and differently from Buttelmann and colleagues, we adopted a combination of body-posture and mouse-tracking techniques to study early implicit belief-tracking and motor processes in the adult observer. Nonetheless, since the task by Buttelmann and colleagues is a variation of the classic false belief change-of-location scenario [34] and is rich in bodily kinematics produced by the agent, its procedure is well suited to study how the motor system resolves social situations involving beliefs [26]. Further, although we did not attempt to make any predictions about adults’ final helping choice, it is worth noting that our computerized task retained aspects of the Buttelmann *et al.*’s real-time task that were instrumental to facilitate meaningful comparisons between participants’ hand trajectories directed towards one box or the other. Accordingly, having the agent trying to open the empty box when she knew the object’s true location allowed the analysis of how participants reaching to open the box she was directly struggling with were influenced by her true belief of the object being located inside the other box. On the contrary, if the agent with a true belief had tried to open the box containing the object, this would have probably resulted in participants helping the other agent with trajectories that were not attracted towards the irrelevant opposite box.

**Figure 1.**
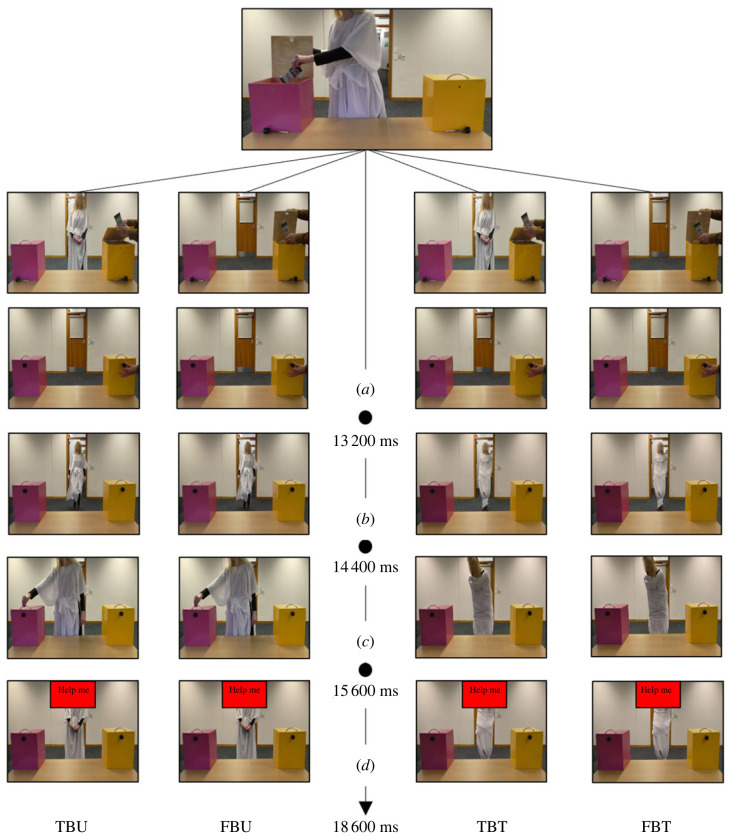
Experiment 1: times of Interest. The balance time window had a fixed duration of 1200 ms starting when the agent came back into the room (*a*) and ending before the agent leaned towards one box (*b*). The mouse tracker time window had a maximum duration of 3000 ms and started when the prompt ‘help me’ appeared on screen (*c*) and ended when the participant clicked on one of the two alternatives (*d*). The face of the confederate agent is blurred only for the purposes of publication.

Four video clips were adopted as experimental stimuli (see figure 1 for a schematic of their time sequence). Each participant watched each experimental stimulus 18 times, for a total of 72 trials. The order of the videos and the initial location of the chocolate was randomized across participants:

(i) **true belief untied** (TBU): after the agent placed a chocolate bar in one of the two boxes, the experimenter (watched by the agent) moved the chocolate bar from that box to the other box. Then the agent left the room. While the agent was outside the room, the experimenter locked the boxes with a black pin. Note that the locking mechanism was seen from the participant’s point of view but not by the agent. Then the agent came back into the room and, after 1200 ms, she reached for the empty box. After unsuccessfully trying to open the lid, the agent assumed a neutral position and the sentence prompt ‘help me’ appeared on the screen;

(ii) **false belief untied** (FBU): after placing a chocolate bar in one of the two boxes, the agent left the room. While the agent was outside, the experimenter moved the chocolate bar from one box to the other and he locked the boxes with a black pin. Note that the locking mechanism was seen from the participant’s point of view but not by the agent. Then the agent came back into the room and, after 1200 ms, she reached for the empty box. After unsuccessfully trying to open the lid, the agent assumed a neutral position and the prompt ‘help me’ appears on the screen;

(iii) **true belief tied** (TBT): after the agent placed a chocolate bar in one of the two boxes, the experimenter (watched by the agent) moved the chocolate bar from that box to the other. Then the agent left the room. While the agent was outside the room, the experimenter locked the boxes with a black pin. The locking mechanism was seen from the participant’s point of view but not by the agent. When the agent came back into the room it was clearly shown that her ability to move was impaired by bandages blocking her arms and legs (the agent’s tunic was warn as movement-restricting bandages). After 1200 ms, the agent leaned towards the empty box. Then the agent assumed a neutral position and the promt ‘help me’ appeared on the screen; and

(iv) **false belief tied** (FBT): after placing a chocolate bar in one of the two boxes, the agent left the room. While the agent was outside, the experimenter moved the chocolate bar from one box to the other and he locked the boxes with a black pin. The locking mechanism was seen from the participant’s point of view but not by the agent. Then the agent came back into the room and it was clearly shown that her ability to move was impaired by bandages blocking her arms and legs. After 1200 ms, the agent leaned towards the empty box. Then the agent assumed a neutral position and the prompt ‘help me’ appeared on the screen.

Participants’ leaning on the WBB was recorded during a time window with a fixed duration of 1200 ms starting when the agent came back into the room and ending before the agent leaned towards one of the boxes. The mouse tracker time window had a maximum duration of 3000 ms and started when the prompt ‘help me’ appeared on screen and ended when the participant clicked on one of the two alternatives (figure 1). Participants were instructed to start moving the mouse as soon as possible and, if they took 400 ms or longer to move their mouse a message appeared on screen prompting a faster response in the following trials (i.e. ‘please start moving earlier on, even if you are not fully certain of a response yet’). If they took more than 3000 ms to click, a warning message appeared on screen (i.e. 'time out!').

#### 2.1.3. Apparatus and measures

The WBB (Nintendo, Kyoto, Japan) is a force platform included in the popular game Nintendo WiiFit. The WBB has been proven to be a reliable tool measuring temporally and spatially sensible information about the body’s centre of pressure (COP) [35]. We used the WBB to measure participants’ anticipatory mediolateral shifts in balance posture exhibiting a lean towards the right-side box or towards the left-side box during the time of interest by calculating the average participants’ COP displacement from the COP position at the beginning of the time of interest. The WBB was connected to a computer via Bluetooth and custom software (provided by Nathan van der Stoep at https://www.multisensoryspacelab.com/) was used to calibrate participants’ baseline COP and to record their shifts in COP during the time of interest. The WBB data was post-processed by resampling at a stable 50 Hz and processed through an eight order Butterworth filter with a low-pass set at 12 Hz, as suggested by Clark *et al.* [35].

Freeman and Ambady’s Mouse Tracker software [36] was used to record participant’s final helping behaviour as a binary outcome (i.e. participants either clicked on the now-full box or the now-empty box), and to analyse the mouse cursor trajectories. Typically, in a mouse-tracker experiment, participants begin a trial by clicking a start box located in the bottom centre of the screen and then they move the mouse cursor to one of two alternatives in the top corners of the screen. The resulting trajectory is recorded at a high temporal resolution of 60–75 Hz [36] and provides information about the attraction that the unchosen alternative has on the participant. Such mouse trajectory attraction is commonly operationalized in terms of area under the curve (AUC; the geometrical area between the ideal trajectory and the participant’s trajectory). The AUC is calculated by summing any curvature heading towards the unchosen alternative (computed as positive AUC) and any curvature heading away from the unchosen alternative (computed as negative AUC) [37,38]. For example, when participants are asked to categorize a face as belonging to a male or female, the AUC of their mouse trajectories is greater when they are shown a picture of a male with feminine features (e.g. long hair) compared to when they are presented with a picture of a male with typical male features [39]. The responses were recorded with a Logitech G502 mouse (set at 1000 dpi and 125 Hz). The two clickable response boxes were over-imposed on the unlocking mechanisms, which were 56 pixels in height and width and were located approximately on the midline of the screen (i.e. 600 pixels upwards and 1690 pixels sideway).

### 2.2. Results

#### 2.2.1. Helping behaviour analysis

Because the final helping behaviour had a binary outcome (i.e. participants had to help the agent by opening either the now-full box or the now-empty box) that was recorded in multiple trials, we calculated the final helping behaviour proportions. The final helping behaviour proportion was defined as the number of choices to help with the now-full box in a specific condition (e.g. TBU) divided by the total number of the trials in that condition. As the proportions were not normally distributed, we performed all the analysis with non-parametric tests (Wilcoxon signed-rank tests).

The results showed that participants chose to help with the now-full box significantly more in the FBU condition compared to the TBU condition (*Z* = −5.012, *p* < 0.001), more in the FBT condition compared to the TBT condition (*Z* = −3.104, *p* = 0.002), and more in the FBU condition compared to the FBT condition (*Z* = −4.336, *p* < 0.001). No difference in helping behaviour was detected between TBU and TBT conditions (*Z* = −0.016, *p* = 0.987) (table 1).

**Table 1.** Experiment 1: descriptive statistics of the proportions of final helping behaviour with now-full box as outcome by condition (TBU, true belief untied; FBU, false belief untied; TBT, true belief tied; FBT, false belief tied).

	mean	median	s.d.
TBU	0.63	0.83	0.40
FBU	0.97	1.00	0.06
TBT	0.63	0.78	0.38
FBT	0.80	0.94	0.31

#### 2.2.2. Anticipatory leaning analysis

**Figure 2.**
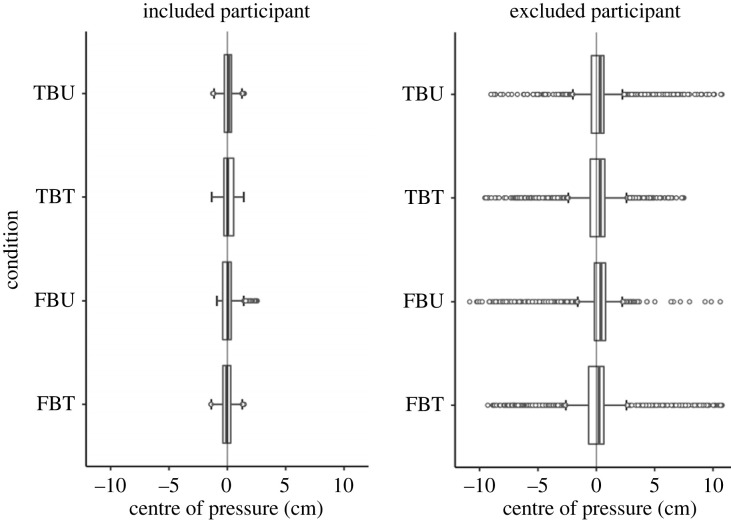
Experiment 1: example of displacement from body midline of one included participant compared to one excluded participant. Positive values reflect rightward body shifts; negative values reflect leftward body shifts. The continuous line in the box represents the median, length of the box represents the interquartile range (IQR) and the whiskers extend to the highest and lowest observations. Points outside the box are outliers (quartile ±1.5 times the IQR).

We analysed participants’ mediolateral leaning on the WBB (*n* = 46/57). Given the multi-trial nature of experiment 1, some of the participants found it difficult to remain still and relaxed for the whole duration of the experiment. For this reason, we visually inspected the raw WBB data to check for participants’ ability to consistently hold a relaxed and stable body posture. In line with the approach used by Zwaan *et al.* [40], we made sure to analyse mediolateral leaning of participants who were able to keep an adequate neutral body posture throughout the duration of the experiment. Participants (11) with a COP exceeding ±4 cm before or during the critical time-window were excluded (see figure 2 for an example of the distribution of body displacement in one included participant versus one excluded participant). Mann–Whitney *U*-tests were conducted to determine whether there was a difference in the average leaning between conditions.

**Figure 3.**
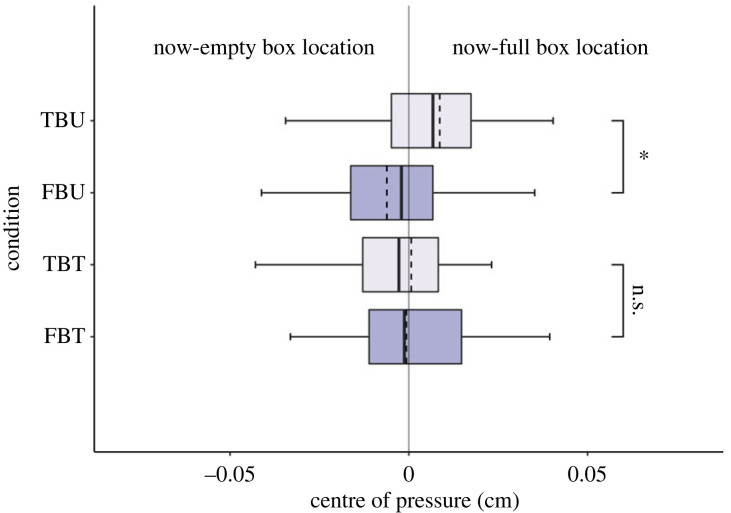
Experiment 1: displacement from body midline. Displacement from body midline (0) between groups (TBU, true belief untied; FBU, false belief untied; TBT, true belief tied; FBT, false belief tied). Positive values reflect a leaning towards the now-full box; negative values reflect a leaning towards the now-empty box. The dotted line in the box represents the mean, continuous line represents the median, length of the box represents the interquartile range and the whiskers extend to the highest and lowest observations.

In line with Zani *et al.* [26], results revealed a significant difference between TBU and FBU conditions (Mann–Whitney *U* = 748, *p* = 0.015; figure 3), with participants in the FBU condition leaning towards the now-empty box (*M* = −0.007, Mdn = −0.002, s.d. = 0.03, confidence interval (CI) = −0.015, 0.003) and participants in the TBU condition leaning towards the now-full box (*M* = 0.007, Mdn = 0.004, s.d. = 0.026, CI = −0.001, 0.014). Instead, when the agent was constrained, anticipatory leaning in true versus false belief conditions was not significantly different: TBT-FBT (Mann–Whitney *U* = 1053, *p* = 0.969) with participants’ body posture almost overlapping across tied conditions (TBT: *M* = 0.002, Mdn = −0.003, *s*.*d*. = 0.04, CI = −0.01, 0.014; FBT: *M* = −0.003, Mdn = −0.002, s.d. = 0.031, CI = −0.012, 0.006). Non-significant differences emerged from the remaining Mann–Whitney *U* tests conducted between TBU-TBT (Mann–Whitney *U* = 846, *p* = 0.098) and FBU-FBT (Mann–Whitney *U* = 968, *p* = 0.482).

#### 2.2.3. Mouse-tracking analysis

In the following data analysis, we explored the amount of attraction exerted by the non-chosen alternative on the trajectories of the mouse cursor, as expressed by the AUC of each trajectory.

Before performing formal analysis, we excluded from the dataset all trials (4.45%) in which the initiation time (IT; amount of time that the mouse cursor takes to reach a distance of 30 pixels from the centre of the starting location) was more than 400 ms. Having an IT set at 400 ms is not necessary when running a Mouse Tracker experiment, and some researchers avoid using it altogether (e.g. [41,42]); nonetheless, it has been described as the optimal cut-off to be adopted when measuring online processes in Mouse Tracker experiments (e.g. [43,44]). Since all participants had an IT below 400 ms in more than 75% of the trials across and within conditions, they were all included in the final analysis.

After checking the raw data for an IT of 400 ms or longer, as per standard practice in mouse-tracker experiments (e.g. [36,45,46]), we remapped all trajectories to one side of the screen and, because raw trajectories vary in duration (and thus they contain a different number of data points), we normalized them into 101 time steps using the linear interpolation provided in the Mouse Tracker Analyser software [43]. Combined, these two transformations facilitate meaningful comparisons. Lastly, we excluded trials in which the AUC was deviating more than 2 s.d. from the average.

Several participants did not choose to click the now-full box and the now-empty box at least once per condition (e.g. multiple participants never clicked the now-empty box in the FBU condition). For this reason, similar to the approach adopted by Kieslich & Hibig [42], we analysed Mouse Tracker data in a linear-mixed model using participants as random intercept and final choice (now-empty; now-full), condition (true belief; false belief), constraint (untied; tied) and their interaction as predictors. Table 2 provides the descriptive statistics about the number of times (i.e. ‘count’) that participants chose to help by clicking on one box (e.g. now-empty; ne-) or the other (e.g. now-full; nf-) in each condition.

**Table 2.** Experiment 1: mean areas under the curve (AUC) by chosen box in each condition (neTBU, now-empty box in true belief untied; nfTBU, now-full box in true belief untied; neFBU, now-empty box in true belief untied; nfFBU, now-full box in false belief untied; neTBT, now-empty box in true belief tied; nfTBT, now-full box in true belief tied; neFBT, now-empty box in false belief tied; nfFBT, now-full box in false belief tied).

	count	mean AUC	s.d.
neTBU	348	−0.066	0.343
nfTBU	606	−0.019	0.356
neFBU	161	−0.011	0.257
nfFBU	785	−0.001	0.167
neTBT	359	−0.007	0.274
nfTBT	596	−0.005	0.154
neFBT	190	−0.019	0.254
nfFBT	768	−0.010	0.227

No significant main effects nor interactions emerged. No main effect of choice (*F*_1,335_ = 0.534, *p* = 0.465), condition (*F*_1,335_ = 0.800, *p* = 0.372) nor constraint (*F*_1,335_ = 0.074, *p* = 0.786); no interaction between condition and choice (*F*_1,335_ = 1.046, *p* = 0.307), between condition and constraint (*F*_1,335_ = 2.181, *p* = 0.141), between choice and constraint (*F*_1,335_ = 0.104, *p* = 0.747) nor between condition, choice and constrain (*F*_1,335_ = 1.140, *p* = 0.286).

In general, unlike van der Wel *et al.* [25], participants’ trajectories were not attracted towards the unchosen alternative, as indicated by negative AUC values.

### 2.3. Discussion

Considering that there is no normatively correct helping response to Buttelmann’s helping task [47], we did not make any specific prediction on participants’ final helping behaviour. Nonetheless, adults showed a pattern of explicit behaviour that could be interpreted as similar to the results found by the original authors. In fact, although participants in experiment 1 chose to help by clicking the now-full box more than the now-empty box in all the conditions, they did so more in FBU compared to the TBU as well as more in the FBT compared to the TBT. One possibility, as per Buttelmann and colleagues’ explanation [33], is that participants helped in retrieving the object more in the false belief conditions because they understood that the agent was trying to retrieve the chocolate where she last saw it, so they helped her in getting the chocolate. And they opened the empty box more in the true belief condition because they reasoned that since the agent knew where the object was she must have had another reason to try to open the empty box. Alternatively, it might be that secretly hiding the chocolate in the false belief condition made it more salient so to generate the expectation that the agent was going to look for it [48].

The analysis of adults’ mediolateral balance shifts, in line with the results obtained by Zani *et al.* [26], confirmed spontaneous motor anticipation of belief-based actions in FBU and TBU conditions. In the FBU condition, adults leaned towards the empty box, i.e. the box in which the agent believed the object to be located; in the TBU condition, they leaned towards the full box i.e. the box in which the agent knew the object was located. The fact that the mediolateral difference between true belief condition and false belief condition disappears when manipulating the agent’s ability to move (i.e. TBT = FBT) is suggestive of an attenuation of participants’ ability to motorically represent the goal of the observed action. However, the lack of a significant effect within conditions (i.e. TBU = TBT; FBU = FBT) also indicates that the effect of constraint on the ability to generate motor predictions about observed belief-based actions is not conclusive. One potential explanation for why anticipatory mediolateral leaning was not completely obliterated by motor restrictions is that constraining the agent might not be as effective in disrupting motor processes in the observer as directly interfering with participants’ ability to move [31], or as disrupting their motor-related cortical areas [49]. However, impairing an agent’s ability to move has been shown to be effective in disrupting motor preparation [32] in the observer. Alternatively, it may be that the motor system might not be necessary for fast action prediction, as indicated by evidence showing that participants born without upper limbs are able to generate behavioural expectations [50]. Another possibility is that the motion of a constrained agent who consistently leans towards one box before asking for help triggers motor representations in the observer. In fact, although such motion occurred after the WBB time window, the participants’ motor system might have used this information to generate expectations about which location the constrained agent was going to lean towards in the upcoming trials. Overall, more research is needed to clarify the boundaries and limits of spontaneous action prediction.

Finally, in contrast with the effect observed by van der Wel and colleagues [25], participants’ mouse cursor trajectories did not reveal conflicts between one’s own and the agent’s beliefs. One possible explanation is that mouse trajectories, similar to other implicit measures for studying belief tracking may be unstable and difficult to replicate [51]. Alternatively, the lack of attraction towards the box in which the agent believed the chocolate to be located might have originated from confounds that were introduced in the design phase. In our attempt to maintain as much as possible some of the ecological validity of the original Buttelmann *et al.*’s helping task [33] as well as of Zani *et al.*’s task [26], we asked participants to click on response boxes that were over-imposed (i.e. camouflaged) onto the unlocking mechanism. The fact that the response boxes were not in the top right and top left corners, as it is commonly done in mouse tracker experiments (e.g. [25,52]), but on the midline of the screen, might have forced participants in experiment 1 to exert an early correction towards the chosen alternative. This could have ultimately resulted in trajectories not having the time and space to be pulled towards the opposite location. In addition, it may be that participants were not as much motivated in following the sequence of events as in van der Wel *et al.* [25], or that they did not visually perceive the agent at the crucial response time. Participants may have decided how they would respond to the upcoming request for help well before it appeared; for instance, perhaps seeing the object’s final location triggered a decision about where to help and ignored any events occurring after this. In the same vein, they may have learned that if the chocolate was initially located in one box, they were going to have to click the other box because the location was always swapped. In this case, participant could have lost any motivation to spectate the scene, even before the belief induction phase.

## 3. Experiment 2

We originally adapted Buttelmann *et al.*’s real-time interaction task [33] for experiment 1 because of its potential for documenting spontaneous motor processing of others’ belief-based actions. Our computer-based task was successful in eliciting participant’s anticipation of the actions of an agent who was free to move. However, bodily constraining the agent was only partially effective in disrupting participants’ motor representations, and the mouse-cursor trajectories they produced were not influenced by the agent’s beliefs. Experiment 2 was conceived to overcome the design limitations of experiment 1 while maintaining the aim of improving our understanding of the functional relationship between motor processes and belief-tracking.

Considering the design limitations of using a computerized version of the helping task to study fast mindreading and motor processes, participants in experiment 2 were not given the possibility to help the agent with either the now-empty box or the now-full box. Instead, they were instructed to click on the final location of the chocolate. In particular, removing the helping component conferred three advantages. First, not having the agent leaning towards one box before asking for help removed the motoric confound of a constrained agent who retains some degree of freedom to move. Second, asking participants to indicate the location of the chocolate allowed using response boxes with the standard size and location found in the literature (e.g. [25,52]), as opposed to the more ecologically valid (but small and awkward in location) camouflaged response boxes adopted in experiment 1. Third, by having correct–incorrect responses (as per van der Wel *et al.* [25]), we were able to discard the wrong responses so to isolate the effect of the agent’s irrelevant beliefs and her ability to move on mouse cursor trajectories landing on the actual location of the chocolate. In addition, a go/no-go manipulation was also introduced in experiment 2. We exploited the fact that when go/no-go stimuli are presented in a fixed location, participants’ visual attention is selectively deployed in anticipation of the forthcoming stimuli [53]. To this end, we ensured that participants paid attention throughout all the duration of the trials and that they visually perceived the agent by instructing them to provide a response at the end of the sequence of events only if the agent was present on the scene.

Overall, experiment 2 was designed to test if adults’ automatic belief tracking abilities (as revealed by their mouse cursor trajectories) are modulated when the agent is physically constrained as opposed to when she is able to move. It is worth noting that the data collection was performed in New Zealand during a period in which COVID-19 rules were temporarily lifted. We were required to design experiment 2 as a computer-based task that could be run on multiple (i.e. 14) computers at the same time to allow fast data collection. Although there are disadvantages to group testing compared to individual testing, such as an increased difficulty in the control of the testing environment [54], an experimenter was always present to monitor that the session was proceeding appropriately (no intervention was ever required) and we used no-go trials to ensure that participants looked at their screen and were focused on their task.’

### 3.1. Method

#### 3.1.1. Participants

G* Power 3.1 indicated that a sample size of 35 would be needed to reach the desired power of 0.80 for the style of data characteristics used by van der Wel *et al.* [25] (input: *f* (*U*) = 0.33, error probability = 0.05; number of groups = 1, number of measurements = 4). Fifty-five right-handed adults were recruited for this experiment in exchange for course credit. Eight participants were excluded because they did not pay attention, as indicated by more than 25% of commission errors (i.e. execution of go responses in no-go trials). The final sample of 47 participants (*M* = 19.4 years, range = 17–31 years, 40 females and seven males) was more than sufficient to reach the desired power of 0.80.

#### 3.1.2. Design and stimuli

Participants were tested in groups of 14 in a single experimental session lasting 20 min. Each participant was assigned to a computer with a blank screen and a start button already set up to initialize the experiment after receiving the instructions. The instructions were provided simultaneously to all participants as follows.

While a video still representing the agent standing between two boxes and holding a chocolate bar was projected on a big screen, the experimenter read out loud the attached caption. ‘This is a computer-task that takes about 10–15 min. For every trial you will see a scene showing two boxes and a person inside a room, and also there is a single chocolate bar being moved from one box to another box. You have to observe what happens. After you hear a beep sound, the person can either come back to the room or not. If the person comes back into the room, you have to click on a button as quickly as possible to indicate whether the chocolate bar is in the right box or the left box. If the person does not come back into the room, you don’t have to click on the button and the scene will end on its own. Let me show you what a trial looks like. After signing the consent form, put your headphones on and click OK to start the computer-task.’ At this point, a true belief example trial was played for everyone to see, then participants could begin the experiment.

**Figure 4.**
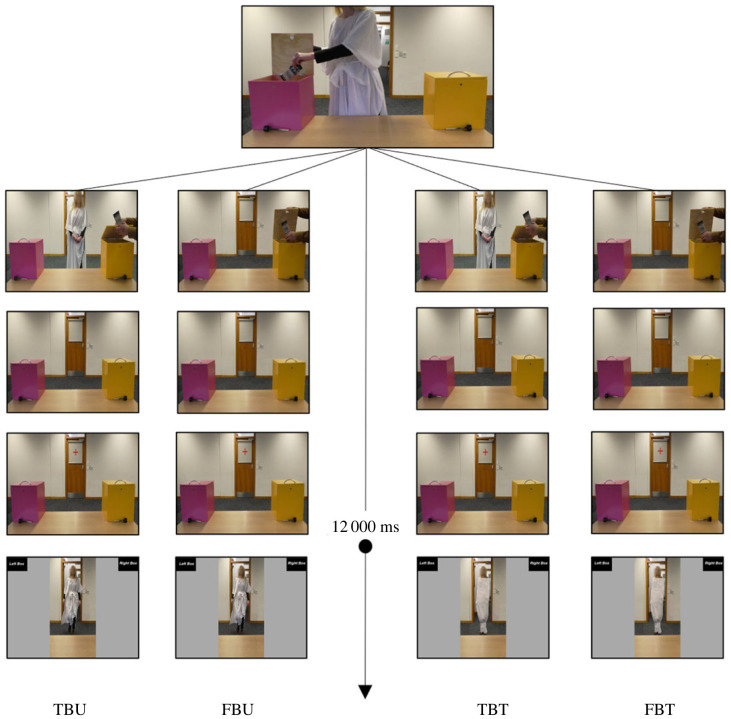
Experiment 2: times of events. Schematic of the relevant events occurring in the true belief untied (TBU), false belief untied (FBU), true belief tied (TBT), and false belief tied (FBT) conditions. After 12 000 ms, the agent came back into the room and participants had to click on the top right or top left corner of the screen to indicate the location of the chocolate. The face of the confederate agent is blurred only for the purposes of publication.

Four video clips were adopted as experimental stimuli (see figure 4 for a schematic of the crucial events as they occurred in each condition). The videos were presented in eight blocks of four videos each, for a total of 32 trials. In addition, one no-go trial for each block was added to ensure that participants were motivated to watch the videos and were visually processing the agent in the go trials. The order of the videos and the location of the chocolate was randomized across participants. If participants took 400 ms or longer to move their mouse, after their choice, a message appeared on screen prompting a faster response in the following trials (i.e. ‘please start moving earlier on, even if you are not fully certain of a response yet’) and, if they took more than 3000 ms to click, a warning message appeared on screen (i.e. ‘time out!’). Further, if participants clicked on the wrong location (i.e. the empty box), the message ‘wrong location’ appeared on screen, and if they clicked when they were not supposed to (i.e. in the no-go trials) they were shown the message ‘click only if the woman is in the room’.

#### 3.1.3. Apparatus and measures

The experimental room was 880 cm wide, 1500 cm long and 250 cm tall. The curtains were always drawn and a standard light attached to the ceiling at the centre of the room was turned on and was not reflecting on any computer screen. Seven participants sat at individual desks facing the left long side of the room and the remaining seven participants sat facing the right side of the room. The distance between the two rows of participants was 400 cm back-to-back while the distance between participants in the same row was of 150 cm head-to-head. An experimenter sat between the two rows to monitor the session. Participants’ responses were recorded with the commercial mice Dell MS116 (1000 Hz, set on the standard medium speed of Windows 10). The mouse-tracking software response boxes were located in the top corners of the screen and were 150 pixels in height and 300 pixels in width. The audio level was set on level 8 of Windows 10 on all computers.

### 3.2. Results

#### 3.2.1. Mouse-tracking analysis

Before performing formal analysis, we excluded from the dataset all trials (12.04%) in which the IT was longer than 400 ms. Furthermore, similar to Wirth *et al.* [55], all trials (3.27%) in which participants reached and clicked the wrong box (i.e. the now-empty box) were counted as errors and discarded. Participants (eight) with more than 25% of commission errors (i.e. execution of go responses in no-go trials) were excluded from all data analysis.

All participants included in the final data analysis (47/55) had an IT below 400 ms in more than 75% of the trials across and within conditions, they had an aggregate of 10.63% of commission errors and they showed a good understanding of the instructions by committing less than 5% of wrong box errors.

After checking the raw data for an IT of 400 ms or more, go/no-go performance and errors, as per standard practice in mouse-tracker experiments (e.g. [36,45,46]), we remapped all trajectories to one side of the screen and we normalized them into 101 time steps using the linear interpolation provided in the Mouse Tracker Analyser software [43]. Finally, we excluded trials in which the AUC was deviating more than 2 s.d. from the average of all trials of that condition.

**Figure 5.**
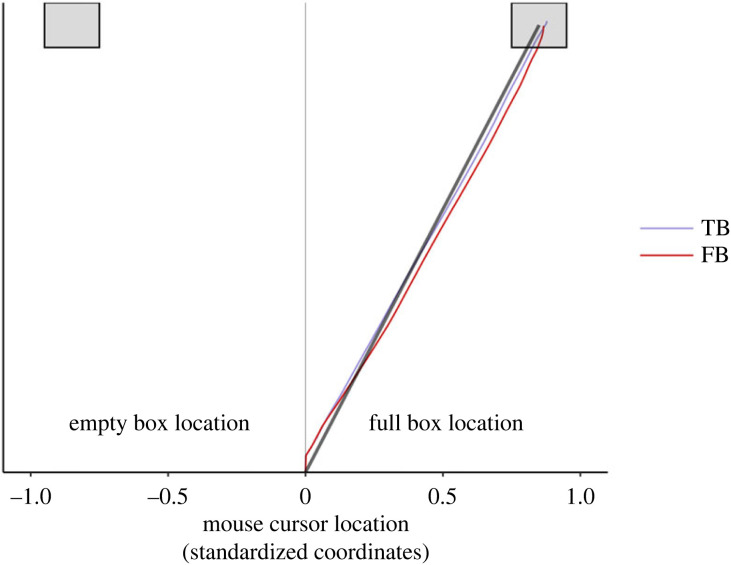
Experiment 2: comparison between true belief (TB) and false belief (FB) trajectories. Visual representation of averaged mouse cursor trajectories in the TB and FB conditions (with the factor constraint collapsed). The straight black line represents the ideal path connecting the starting location with the full box. Participants’ trajectories in the FB condition were more attracted towards the box containing the object (or more repulsed by the box in which the agent falsely believed the object was located.

A linear-mixed model with an unstructured within-subject error covariance using participants as random intercept and including belief (true belief, false belief) and constraint (untied, tied) as fixed factors showed a significant effect on AUC of belief (*F*_1,46_ = 8.270, *p* = 0.006). Participants clicked the box containing the chocolate with hand trajectories that were more similar to the ideal straight line connecting the starting location to the target box in the true belief condition compared to the false belief condition. This is indicated by AUCs being closer to zero in TBU and TBT compared to the more negative AUCs of FBU and FBT conditions (figure 5). Recall that an AUC equals to zero when the trajectory does not curve towards to, nor away from the unchosen alternative (i.e. ideal trajectory); it is positive when it is attracted towards the unchosen alternative, and it is negative when it is repulsed away from the unchosen alternative. There was no significant effect of constraint (*F*_1,46_ = 0.307, *p* = 0.582) nor interaction between belief and constraint (*F*_1,46_ = 0.067, *p* = 0.797) (see descriptive statistics in table 3).

**Table 3.** Experiment 2: mean areas under the curve (AUC) of participants’ mouse tracker trajectories in true belief untied (TBU), true belief tied (TBT), false belief untied (FBU), and false belief tied (FBT) conditions. The more AUC is negative, the more the trajectory is attracted towards the chosen alternative.

	mean AUC	s.d.	*n*
TBU	−0.031	0.139	47
TBT	−0.036	0.197	47
FBU	−0.070	0.149	47
FBT	−0.083	0.176	47

### 3.3. Discussion

As of today, the only published study using mouse-tracker technology to investigate mindreading processes indicates that adults click the location of a ball with hand trajectories that are influenced by where an agent believes the ball to be [25]. Since experiment 1 did not detect any effect of the agent’s belief on the mouse cursor trajectories of participants who clicked to help, modifications to the experimental design were implemented in experiment 2.

In experiment 2, to ensure that participants paid attention to the scene and that they visually perceived the agent during the crucial time window, we implemented a go/no-go manipulation requiring participants to click the location of the chocolate when the agent came back into the room and to withhold their response when the agent did not come back into the room. Similar to van der Wel *et al.*, we predicted that mouse cursor trajectories would have been more attracted towards the empty box when the agent falsely believed the chocolate to be stored in the empty box compared to when the agent knew that the chocolate was in the full box. In addition, we expected that impairing the agent’s ability to move would have also impaired participants’ fast belief tracking abilities. However, constraining the agent did not influence movement parameters, and the main effect of belief had a puzzling opposite direction to what we expected, with participants’ trajectories generally (i.e. regardless of the constraint) being pulled in the opposite direction of the empty box when the agent falsely believed that the chocolate was in the empty box. One possible explanation is that participants’ hand movements were generally reaching the target by following the shortest path and that the main effect of belief was the result of some noise in the data. Alternatively, it might be that participants in the false belief condition had to counterbalance their tendency to move towards the empty box to avoid an incorrect response, paradoxically resulting in them having trajectories more attracted towards the full box. In the true belief condition, the instruction of clicking the box containing the chocolate combined with the agent’s true belief did not generate the same conflict, and trajectories were closer to be ideal. Indeed, it has been suggested that sometimes the suppression of a pattern of motor activity is a mechanism that comes into place to prevent the urge to provide a non-required response [56] and that the more motor processes for an action are active, the more those processes are subsequently inhibited [57]. In experiment 2, participants were instructed to watch the unfolding of the events and to click the location of the chocolate, so it is possible to conjecture that a quick and prepotent response to move the mouse cursor towards the (belief-congruent) empty box was prepared, and adjustments took place to prevent a wrong behaviour.

## 4. General discussion

Reasoning and deliberating about others’ actions is an ordinary ability that we all have first hand experience with. However, how humans sustain fluid social interactions in real-time by accurately anticipating what others are going to do is far from clear. Here, we tested adult participants to investigate the possibility that, since motor processes can spontaneously guide how the outcomes of an action are predicted (e.g. [17]), it is conceivable—where that kind of goal ascription occurs in false-belief tasks—for motor representations to account for someone’s belief-like state [19]. The idea here was that, by using the efficiency of the motor system, mindreading could be achieved with a minimal cognitive cost by feeding belief-related information into motor representations and without reasoning about beliefs as such. Two experiments extended on the initial evidence showing that an agent’s belief-like state can modulate motor representations in the observer [26], and measured whether motor processes contribute to the use of information about beliefs when observers spontaneously anticipate another’s actions.

In support of a tight relationship between belief-tracking and motor processes, our anticipatory leaning results indicate a reliable influence of the agent’s belief on mediolateral balance in the untied condition but not in the tied condition. In experiment 1, participants spontaneously shifted their balance towards the empty box in the false belief condition and towards the full box in the true belief condition when the agent was free to move. Crucially, such belief-congruent modulation of the motor representations of a goal-directed action was attenuated when the agent was motorically constrained, as indicated by lack of anticipatory leaning. That is, WBB results suggest that the agent’s inability to move prevented adult observers to generate motor representations of an action, and information about beliefs-like states lacked a framework (at least a motoric one) to express itself. Experiment 1 also revealed a pattern of adults’ explicit helping behaviour that is consistent with Buttelmann *et al.*’s [33] original findings with young children. Participants chose to help with the full box more in the false belief condition compared to the true belief condition when the agent was free to move. Interestingly, the same difference between false and true belief conditions emerged when the agent was constrained. Then, if we accept Buttelmann *et al.*'s interpretation that this pattern of results is suggestive of participants holding the agent’s belief into consideration when providing explicit responses, the WBB and the explicit behaviour results combined are in line with the conjecture that impairing an observer’s ability to motorically represent the outcome to which a belief-based action is directed towards would negatively impact spontaneous mindreading while sparing flexible reasoning about others’ beliefs.

In terms of mouse cursor trajectories, we did not detect any evidence of online conflicts between participants’ and agent’s beliefs in experiment 1, and experiment 2 had puzzling results. In experiment 1, regardless of the condition, hand movement parameters revealed that choice selection was performed with confidence by reaching the target alongside an ideal straight trajectory connecting the starting location with the chosen box. In experiment 2, contrary to our predictions and differently from van der Wel *et al.* [25], mouse trajectories landing on the location of the object were not curved towards but away from the location in which the agent falsely believed contained the object. There are two possible explanations. One possibility is that hand movement parameters, contrary to how participants’ body posture is influenced by the agent’s beliefs, were not influenced by the agent’s beliefs. This could be interpreted as an indication that belief-tracking is not cognitively efficient [58], or even that it does not exist at all (e.g. [29,30]), especially if we consider a process to be cognitively efficient only when it is automatic, stimulus-driven and completely independent from cognitive resources. Also, while there is evidence that tracking another person’s belief can be done rapidly and involuntarily (e.g. [25,59]) under cognitive load [60], other authors find that secondary tasks [58] and emotional states [61] can cause a cognitive turbulence that affects even the simplest forms of implicit mindreading. It might be more appropriate to think of efficient, minimal, mindreading as spontaneous rather than fully fledged automatic: its functioning might be largely characterized by fast and unconscious processing of the scene.

There is a second possibility of explaining the mouse cursor trajectories. In experiment 2, we facilitated spontaneous processing of the agent’s belief-like state by asking participants to click on the location of the object only if the agent came back into the room at the end of the sequence of events. That is, using a go/no-go manipulation we ensured that participants deployed attentional resources to the belief induction phase as well as to whether the agent was free to move or constrained. While constraining the agent had no effect on the way participants moved the mouse cursor to the target object, a puzzling effect emerged when considering how participants processed the other person’s beliefs. Different from our prediction, the mouse cursor trajectories were not more attracted but more repulsed by the location where the agent falsely believed the object to be located. One possibility—though not a conclusive one—is that motor representations for the other person’s expected action (i.e. that she will go to the box she believes contains the object) were not more active but inhibited. An observed action usually has a facilitatory rather than inhibitory effect on the motor representations of that action. Nonetheless, a few studies using electroencephalography (EEG; e.g. [57]), transcranial magnetic stimulation (TMS; [62,63]) or a combination of EEG and TMS (e.g. [64]) have suggested that it is possible for a post-stimulus rebound effect to reflect inhibition following the activation of the motor system. Think about a funambulist who is trying to keep an appropriate balance on a rope. Just when he is about to fall on his right, he counters the not-anymore required (if not dangerous) rightward movement by moving leftwards. However, while inhibiting the rightwards movement using a counter movement to his left, he can overdo it, ending up rebounding on the left side of the rope. This rebound reflects inhibition of a previously activated—and ‘now’ unnecessary—representation of an action and may be related to the previous activation of the motor system. When the observed action is relevant to the observer, such as when participants are anticipating what the agent will do, the motor system becomes more activated for a movement that reflects the likely outcome of the observed action (as we found in experiment 1’s anticipatory leaning towards the empty box). Subsequently, such as when the agent’s goal is irrelevant to participants that are instructed to click on the location where they know the object is (i.e. experiment 2), it becomes more inhibited. Regardless, the mouse-tracking results were unlike those obtained by van de Wel *et al.* [25] in a conceptually similar task: participants’ trajectories were not attracted towards the box in which an agent who was free to move believed the object to be located. Since the mouse tracker did not produce the predicted belief congruent compatibility effect in terms of curvature attraction towards the box in which the agent—truly or falsely—believed the object to be located, a post-stimulus rebound interpretation remains a possibility that needs further research and we urge caution in the interpretation of the mouse tracking results.

In conclusion, our results suggest that information about beliefs can be efficiently processed by the motor system to generate accurate behavioural expectations [26]. However, our claim is not that belief-tracking relies exclusively on motor processes. On the contrary, the evidence points in the direction of multiple routes available to mindreading [65] and the data we were able to gather in terms of whether or not motor disruptions negatively impact mindreading is preliminary and leaves the door open to alternative interpretations regarding how exactly belief-related information feeds into motor representations. On the one hand, we found some indications that motor processes and representations in the observer can carry information about beliefs during action observation, and that the way anticipatory leaning is sensitive to belief-based actions can be modulated by disrupting the agent’s possibility to act. On the other hand, the use of the WBB for answering questions about belief-based action understanding is still novel, and the converging evidence we sought by measuring belief-congruent effects on participants’ mouse cursor trajectories warrant further research.

## Data Availability

The Wii Balance software adopted in experiment 1 is freely available upon request at https://www.multisensoryspacelab.com/ and the Mouse Tracker software adopted in experiments 1 and 2 is also freely available at http://www.mousetracker.org/.
